# Correction: Enhancing chronic wound healing with Thai indigenous rice variety, Kaab Dum: Exploring ER stress and senescence inhibition in HaCaT keratinocyte cell line

**DOI:** 10.1371/journal.pone.0306210

**Published:** 2024-06-21

**Authors:** Witchuda Payuhakrit, Pimchanok Panpinyaporn, Wilunplus Khumsri, Gorawit Yusakul, Ratsada Praphasawat, Nitra Nuengchamnong, Sarawoot Palipoch

The fourth author’s name is spelled incorrectly. The correct name is: Gorawit Yusakul. The correct citation is: Payuhakrit W, Panpinyaporn P, Khumsri W, Yusakul G, Praphasawat R, Nuengchamnong N, et al. (2024) Enhancing chronic wound healing with Thai indigenous rice variety, Kaab Dum: Exploring ER stress and senescence inhibition in HaCaT keratinocyte cell line. PLoS ONE 19(5): e0302662. https://doi.org/10.1371/journal.pone.0302662.

## References

[1] Payuhakrit W, Panpinyaporn P, Khumsri W, Yusakul G, Praphasawat R, Nuengchamnong N, et al. (2024) Enhancing chronic wound healing with Thai indigenous rice variety, Kaab Dum: Exploring ER stress and senescence inhibition in HaCaT keratinocyte cell line. PLoS ONE 19(5): e0302662. doi: 10.1371/journal.pone.0302662 38748716 PMC11095683

