# Supplementary material for: Enhancing chronic wound healing with Thai indigenous rice variety, Kaab Dum: Exploring ER stress and senescence inhibition in HaCaT keratinocyte cell line
Source: PLoS One. 2024 May 15;19(5):e0302662. doi: 10.1371/journal.pone.0302662 (PMC11095683; doi:10.1371/journal.pone.0302662)
Supplement: Attachment — Submitted filename: Comments.docx [file pone.0302662.s002.docx]

Dear Prof Dr/Editor,

Greetings!

Thank you for your invitation to review the manuscript entitled ‘’ Enhancing chronic wound healing with Thai indigenous rice variety, Kaab Dum: exploring ER stress and senescence inhibition in HaCaT keratinocyte cell line’’.

Please, see below my comments:

Introduction:

1. Please, explain the relationship between senescence, inflammation, and delayed wound healing.

Materials and methods

2. Please, mention the ethanol: water (V/V) used for your extract.
3. Please, mention the final concentration of your stock in DMSO.
4. Please, mention the solute for MTT solution. Is it medium or PBS?
5. The concentration of MTT is 0.5 mg per L or ml???
6. What is the concentration of DMSO after addition of your extract to the medium???
7. What is the type of fixative you used in SA-β-gal staining assay?
8. Correct the title of Table 1 ………polished and unpolished……..

Results

9. Please, revise the abbreviation of TGF-β in all result section.
10. In the wound healing assay, the image 3 and 4 of 0 h is not similar in size as others.

Discussion

11. The discussion section containing too long paragraphs.
