# Supplementary material for: Enhancing chronic wound healing with Thai indigenous rice variety, Kaab Dum: Exploring ER stress and senescence inhibition in HaCaT keratinocyte cell line
Source: PLoS One. 2024 May 15;19(5):e0302662. doi: 10.1371/journal.pone.0302662 (PMC11095683; doi:10.1371/journal.pone.0302662)
Supplement: Attachment — Submitted filename: Response to reviewers.docx [file pone.0302662.s003.docx]

**Response to reviewers**

PONE-D-23-34554

Enhancing chronic wound healing with Thai indigenous rice variety, Kaab Dum: exploring ER stress and senescence inhibition in HaCaT keratinocyte cell line

PLOS ONE

**Reviewer #1**

| **Comments** | **Responses** |
| --- | --- |
| **Introduction:** 1. Please, explain the relationship between senescence, inflammation, and delayed wound healing. | The relationship between senescence, inflammation, and delayed wound healing was explained and added in the introduction part (paragraph 3 as highlighted text). |
| **Materials and methods** 2. Please, mention the ethanol: water (V/V) used for your extract. | The concentration of ethanol was edited as per the suggestion in line 3 (Rice extraction)  “Ethanol: water 18% v/v” was added as highlighted text. |
| 3. Please, mention the final concentration of your stock in DMSO. | The final stock of rice extract (0.1 g/ml) was indicated in the preparation of the extract as per the suggestion (highlighted text). |
| 4. Please, mention the solute for MTT solution. Is it medium or PBS? | The solute of MTT (media) was mentioned in  MTT assay (highlighted text) as per the suggestion. |
| 5. The concentration of MTT is 0.5 mg per L or ml??? | The concentration of MTT was corrected to  0.5 mg/ml in MTT assay (highlighted text) as per the suggestion. |
| 6. What is the concentration of DMSO after addition of your extract to the medium??? | The stock concentration of rice extract is 0.1 g/ml, and this study used 1 mg/ml as the highest concentration. Therefore, the final concentration of DMSO is equal to 0.1%, which was not cytotoxicity to HaCat cell line. |
| 7. What is the type of fixative you used in SA-β-gal staining assay? | The 2% glutaraldehyde was used and was mentioned in the methodology of SA-β-gal) staining (highlighted text). |
| 8. Correct the title of Table 1 ………polished and unpolished…….. | The title of Table 1 was corrected follow the suggestion (highlighted text). |
| **Results** 9. Please, revise the abbreviation of TGF-β in all result section. | We revised and corrected the abbreviation of TGF-β throughout of the manuscript. |
| 10. In the wound healing assay, the image 3 and 4 of 0 h is not similar in size as others. | We changed the image 3 and 4 of 0 h in Figure 5 to similar in sized as the per suggestion. |
| **Discussion** 11. The discussion section containing too long paragraphs. | We removed some part and re-write the senescence to more concise as per the suggestion. |

**Reviewer #2**: This article presents a study on the therapeutic potential of Kaab Dum rice extract in the context of chronic wound healing. The investigation focused on the effects of the indigenous rice variety on TGF-β-induced HaCaT cells, specifically examining oxidative stress, endoplasmic reticulum (ER) stress, cell cycle arrest, and cellular senescence. The findings suggest a reduction of oxidative stress, inhibition of ER stress, and promotion of wound healing. However, there are areas where the study could be strengthened:

| **Comments** | **Response** |
| --- | --- |
| 1. A more detailed investigation into the mechanisms by which Kaab Dum rice extract affects wound healing is necessary to fully understand its therapeutic potential. | We agree with the suggestion and plan to study the detailed mechanisms of Kaab Dum effect on wound healing for further study. |
| 2. The absence of in vivo studies or animal models is a significant limitation. Such studies are crucial for assessing the physiological relevance of the rice extract in a more complex biological system. | This study is the pioneer in elucidating the cellular mechanism of TGF-β induced delayed wound healing in keratinocytes. Therefore, the in vitro cell line was used. However, we agree with the suggestion and plan to study the physiological relevance of the rice extract in a more complex biological system. |
| 3. Comparing the effectiveness of Kaab Dum rice extract with other treatments for chronic wounds would provide context and further insight. | We agree with the suggestion and plan to study the detailed mechanisms of Kaab Dum effect on wound healing in an animal model for further study and compare with the other treatment per the suggestions. |
| 4. Detailed information about the reagents used in the study should be provided, including the source, stock number, tradename, company, city, and country. This information is critical for the reproducibility of the research. | The details of all reagents were added as per the suggestion (highlighted text). |
| 5. An inconsistency in Figure 5 regarding the cell scratching assay should be addressed to ensure the validity of the results. The scratch area at 0 hr for the groups treated with TGF-β+0.02 mg/ml PKD and TGF-β+0.1 mg/ml PKD does not match the initial scratch area of the other groups. | We change the other representative figures in Figure 5 of those conditions to match the initial scratch area of the other groups as per the suggestion. |
| 6. What is the authors' rationale for choosing TGF-β induced HaCaT cells as a model of chronic wound? What type of chronic wound is this? Further justification and discussion are needed. | The rational of choosing TGF-β induced HaCaT cells as a model of chronic wound was mention in discussion part “TGF-β is focusing in this study because the previous evidence showed TGF-β as the main factor producing chronic wounds, including diabetic ulcers and pressure wounds”as per the suggestion (highlighted text). |

**Reviewer #3**

Dear Authors,
Your manuscript is well-structured and informative. Addressing the following comments will enhance your study's clarity and scientific rigor.

| **Comments** | **Responses** |
| --- | --- |
| 1. Antibiotic-Antimycotic Solution: For clarity, kindly mention the composition of the antibiotic-antimycotic solution used in your experiments. | As per the suggestion, 1% w/v penicillin/streptomycin was mentioned in HaCaT keratinocyte culture (highlighted text). |
| 2. MTT Solution Concentration: Clarify whether the MTT solution concentration of 0.5 mg/L should instead be reported as 0.5 mg/mL for accuracy. | The MTT solution concentration was corrected to 0.5 mg/ml following the suggestion (highlighted text) in MTT assay. |
| 3. Transforming Growth Factor-β (TGF-β): Provide the source and product number of the TGF-β used in your study for better reproducibility. | The source and product number of TGF-β was added (TGF-β: Abbkine, PRP100190) in the DCFH-DA assay, and all reagents also indicate the source and product number (highlighted text). |
| 4. Rice Extract Concentrations: Explain the rationale behind using different concentrations for polished and unpolished Kaab Dum rice extracts (0.02 and 0.1 mg/mL for polished; 0.2 and 1 mg/mL for unpolished). What factors influenced this selection? | The rationale for using different concentrations of rice extract is that we exclude the effect of the cytotoxicity of Kaab Dum rice extracts on the effects of the wound healing process by selecting the non-cytotoxicity of rice extracts. Therefore, 0.02 and 0.1 mg/mL were selected for polished, and 0.2 and 1 mg/mL were selected for unpolished Kaab Dum rice extracts, respectively. |
| 5. Scratch Wound as Chronic Wound: Justify why the scratch wound model was chosen to represent a chronic wound in your study. | The scratch assay is suitable for studies on the effects of cell-matrix and cell-cell interactions on cell migration, which is most affected by the chronic inflammatory environment and causes chronic wounds (PMID: 17406593). Moreover, this assay mimics cell migration during wound healing in vivo and is compatible with imaging of live cells during migration to monitor the molecular mechanism of intracellular events. |
| 6. % of Control and % of Inhibition: Clarify the meaning of "% of control." Does it refer to "% of inhibition"? Please provide clarity on these percentages for better understanding. | The "% of control was used due to the experiments consisted of both induction (TGF-β) and inhibition of oxidative stress value. Therefore % of control was used to normalization of control to 100% for more understand the effects of treatment. In addition we added the oxidative stress represents 100% control in Figure3 legend (highlighted text). |
