# Supplementary material for: Enhancing chronic wound healing with Thai indigenous rice variety, Kaab Dum: Exploring ER stress and senescence inhibition in HaCaT keratinocyte cell line
Source: PLoS One. 2024 May 15;19(5):e0302662. doi: 10.1371/journal.pone.0302662 (PMC11095683; doi:10.1371/journal.pone.0302662)
Supplement: Attachment — Submitted filename: Response to reviewers.docx [file pone.0302662.s004.docx]

**Response to reviewers**

PONE-D-23-34554R1

Enhancing chronic wound healing with Thai indigenous rice variety, Kaab Dum: exploring ER stress and senescence inhibition in HaCaT keratinocyte cell line

PLOS ONE

**Review Comments to the Author**

**Reviewer #2**

| **Comments** | **Responses** |
| --- | --- |
| 1. The LC-ESI-QTOF-MS/MS chromatogram should be marked with the main peak information. | The authors have elucidated the specifics of each peak in the table, so the chromatogram lacks markings of the main peak information. |
| 2. A few typographical errors also remain (i.e. the word “TGF-β” on pg 20-21, Table 2, line 4, [M+Na}? ). | 2.1 While checking our Word file, no typographical errors were detected in the term “TGF-β”, but issues arise when uploading data to the submission system (i.e TGF-🞎).  2.2 Table 2, line 4 was changed “[M+Na}” to “[M+Na]. |
